# Influenza Virus in a Natural Host, the Mallard: Experimental Infection Data

**DOI:** 10.1371/journal.pone.0008935

**Published:** 2010-01-28

**Authors:** Elsa Jourdain, Gunnar Gunnarsson, John Wahlgren, Neus Latorre-Margalef, Caroline Bröjer, Sofie Sahlin, Lovisa Svensson, Jonas Waldenström, Åke Lundkvist, Björn Olsen

**Affiliations:** 1 Section for Zoonotic Ecology and Epidemiology, School of Natural Sciences, Linnaeus University, Kalmar, Sweden; 2 INRA, UR 346, Saint Genès Champanelle, France; 3 Aquatic Biology and Chemistry Group, Kristianstad University, Kristianstad, Sweden; 4 Karolinska Institutet, Microbiology & Tumor Biology Center (MTC), Stockholm, Sweden; 5 Swedish Institute for Infectious Disease Control, Stockholm, Sweden; 6 National Veterinary Institute, Uppsala, Sweden; 7 The Department of Biomedical Sciences and Veterinary Public Health, University of Agricultural Sciences (SLU), Uppsala, Sweden; 8 Section of Infectious Diseases, Department of Medical Sciences, University of Uppsala, Uppsala, Sweden; Centre National de la Recherche Scientifique, France

## Abstract

Wild waterfowl, particularly dabbling ducks such as mallards (*Anas platyrhynchos*), are considered the main reservoir of low-pathogenic avian influenza viruses (LPAIVs). They carry viruses that may evolve and become highly pathogenic for poultry or zoonotic. Understanding the ecology of LPAIVs in these natural hosts is therefore essential. We assessed the clinical response, viral shedding and antibody production of juvenile mallards after intra-esophageal inoculation of two LPAIV subtypes previously isolated from wild congeners. Six ducks, equipped with data loggers that continually monitored body temperature, heart rate and activity, were successively inoculated with an H7N7 LPAI isolate (day 0), the same H7N7 isolate again (day 21) and an H5N2 LPAI isolate (day 35). After the first H7N7 inoculation, the ducks remained alert with no modification of heart rate or activity. However, body temperature transiently increased in four individuals, suggesting that LPAIV strains may have minor clinical effects on their natural hosts. The excretion patterns observed after both re-inoculations differed strongly from those observed after the primary H7N7 inoculation, suggesting that not only homosubtypic but also heterosubtypic immunity exist. Our study suggests that LPAI infection has minor clinically measurable effects on mallards and that mallard ducks are able to mount immunological responses protective against heterologous infections. Because the transmission dynamics of LPAIVs in wild populations is greatly influenced by individual susceptibility and herd immunity, these findings are of high importance. Our study also shows the relevance of using telemetry to monitor disease in animals.

## Introduction

Influenza A viruses (IAVs) have a wide range of host species, including humans, pigs, horses, wild mammals, and birds. Their classification relies on two antigenic surface proteins, the hemagglutinin (HA) and the neuraminidase (NA) for which 16 and 9 different subtypes are known, respectively. Because many combinations of HA and NA have been found in wild waterfowl and the prevalence in these species is high worldwide, they are considered the natural reservoir of IAVs [1]. Prevalence is particularly high in dabbling ducks (i.e. ducks that feed by tipping into the water to graze on aquatic vegetation or feed on small aquatic preys), probably because their feeding behavior favors ingestion of viral particles. In ducks, IAVs usually replicate in the epithelial cells of the intestinal tract and are excreted at high concentrations from the cloaca into water [2]–[4]. The main transmission route in waterfowl is oro-fecal [5] but indirect contamination by ingestion of contaminated water may also play a role in yearly infection dynamics [6]. The peak of IAV isolation occurs during or just prior to the autumn migration, a time when many immunologically naïve juveniles share water with adult birds from different breeding areas [1], [7].

Understanding the ecology of IAVs in their natural hosts is essential for several reasons. First, low pathogenic avian influenza virus (LPAIV) strains of the H5 and H7 subtypes, which are known to circulate in wild birds, may become highly pathogenic (HPAI) if introduced into poultry and cause high morbidity and mortality with severe economic consequences [1]. Second, wild birds represent a reservoir of IAVs that may reassort with human viruses in pigs or other so-called “mixing vessel” hosts and ultimately generate strains with pandemic potential [8]. Finally, it has been suggested that wild birds might be involved in the spread and transmission to humans of IAV strains with direct zoonotic potential [9].

Influenza A virus transmission dynamics in waterfowl may vary depending on whether (1) hosts are affected by infection and (2) herd immunity exists in the population [10]. These two questions were addressed by using an experimental infection approach. The wild type mallard (*Anas platyrhynchos*) was chosen as an experimental model because it is the most widespread and abundant migratory dabbling duck from which IAVs are frequently isolated both in Eurasia and North America [11], [12]. Previous experimental infection studies on mallards or closely related domestic ducks (i.e. Pekin ducks, *Anas platyrhynchos domesticus*) revealed that infection by IAVs generally does not adversely affect this natural host [3], [4], [13]–[18]. However, a recent field study found a lower body mass in mallards positive for IAV compared to negative mallards [19] indicating that there might be an ecological cost of infection despite the absence of obvious clinical signs. Previous successive experimental studies reported the existence of a relative protection against HA homologous [3], [18] and heterologous [18] re-infections.

**Figure 1:**
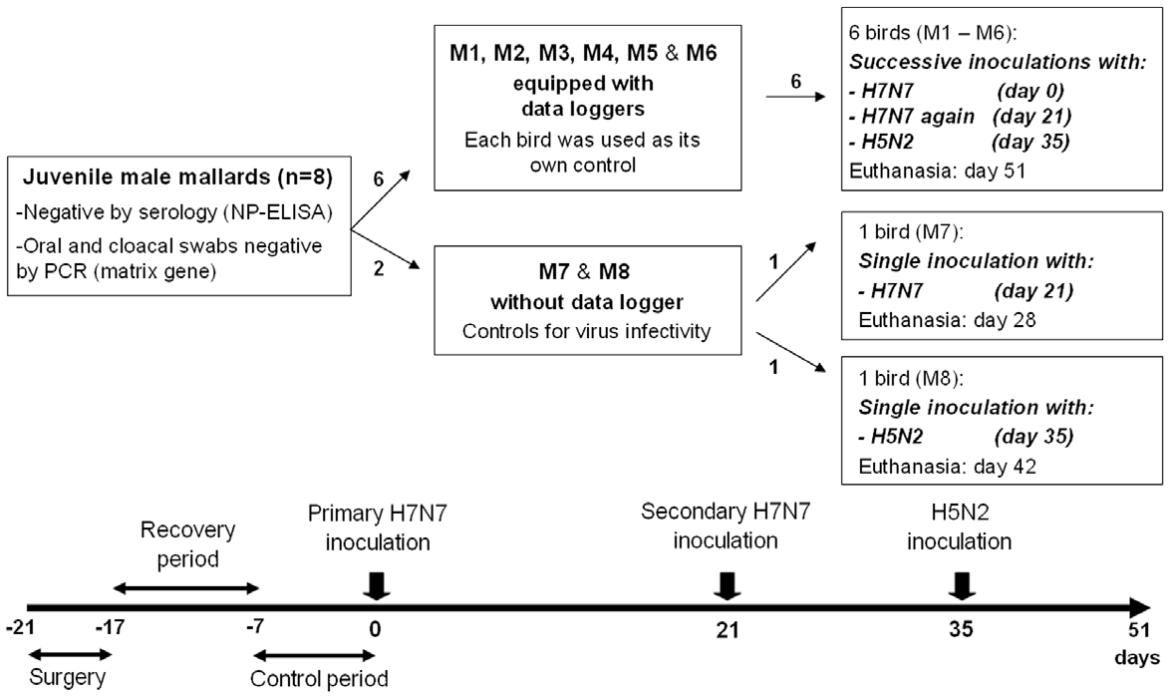
Experimental design.

In this study, we tested the hypotheses that LPAI infection in mallards (1) may have clinical effects on its host; (2) provides short-term homosubtypic immunity (i.e. immunity against re-infection by the same LPAI strain); (3) provides short-term heterosubtypic immunity (i.e. cross-protective immunity to a heterologous LPAIV subtype). Six juvenile male mallards caged individually and continuously monitored by telemetry for body temperature, heart rate, and activity, were successively inoculated with an H7N7 LPAI isolate (day 0), the same H7N7 virus again (day 21) and an H5N2 LPAI isolate (day 35) (Figure 1). We found that birds infected by a LPAIV (1) may develop a slight and transient increase in body temperature, (2) are immune to homosubtypic re-infection and (3) may be immune to heterosubtypic re-infection.

## Results

The six mallards appeared to be unaffected by the successive virus inoculations. They were active, ate and drank normally, and gained weight during the course of the study.

### Telemetry

**Figure 2:**
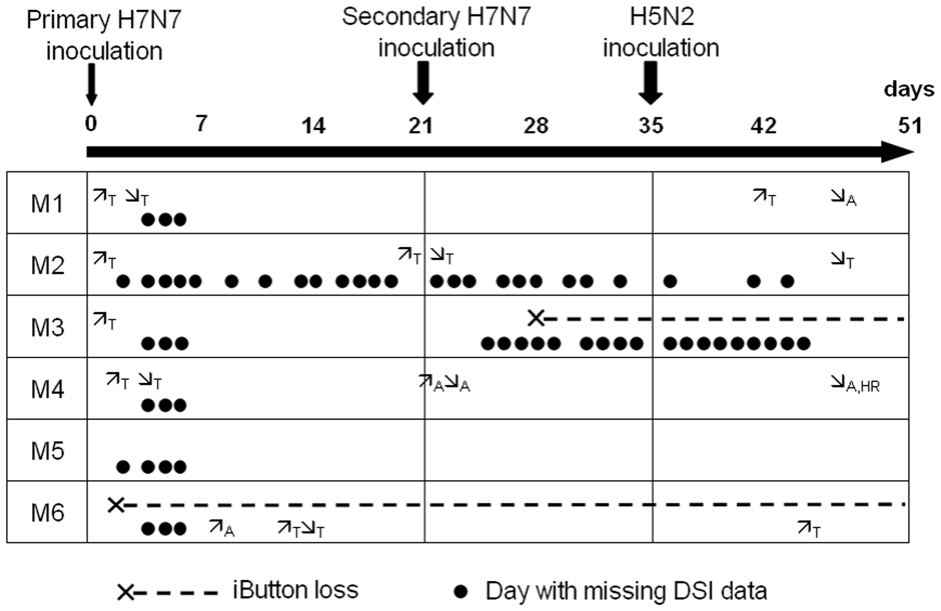
Significant (p<0.05) day to day changes in mean daily body temperature, heart rate and activity recorded for each duck throughout the experiment using General Linear Models and Tukey's post hoc test. For body temperature, when both DSI and iButton data loggers were functional, only the changes detected by both data loggers are indicated.

Baseline values for body temperature, heart rate and activity were recorded for each duck during the control period and used as pre-challenge references (Table 1). After the first H7N7 inoculation, day to day comparisons were performed using General Linear Models (GLMs) and Tukey's post hoc test, each duck being used as its own control. For four ducks (M1-M4), day-to-day comparison showed a significant (p<0.001) increase in body temperature on the day their viral RNA excretion started (Table 2). This increase in body temperature was recorded by both DSI and iButton data loggers (mean = 0.5°C and SD = 0.1°C for both data loggers). A few other significant (p<0.05) day-to-day changes in body temperature, heart rate and activity were recorded but the timing (i.e. days) of these changes was not consistent among ducks (Figure 2).

**Table 1:** Mean individual values recorded by the data loggers throughout the control week.

Duck ID	iButton	DSI	Heart rate	Activity
M1	40.4 (0.2)	40.2 (0.2)	132 (27)	0.8 (2.5)
M2	40.7 (0.2)	40.6 (0.2)	142 (34)	0.7 (1.9)
M3	40.7 (0.3)^a^	40.5 (0.3)	157 (23)	0.4 (1.4)
M4	40.7 (0.2)	40.4 (0.4)	120 (27)	1.4 (4.0)
M5	40.7 (0.2)	40.6 (0.2)	127 (27)	2.4 (4.6)
M6	40.3 (0.5)^b^	40.3 (0.3)	179 (39)	0.6 (1.8)
All birds	40.6 (0.3)	40.2 (0.3)	141 (34)	0.9 (2.7)

Body temperature (DSI transmitter and iButton) is expressed in °C, heart rate in beats per minute and activity in movements per minute. Standard deviation is indicated in parentheses.

a iButton loss on day 28 PI (mean therefore based on data until this date only)

b iButton loss on day 2 PI (mean therefore based on data until this date only)

**Table 2:** Mean daily body temperature values (in °C) recorded by the data loggers after the three successive challenges.

Days after H7N7 primary inoculation		0	1	2	3	21	22	23	24	35	36	37	38
		H7N7 challenge				H7N7 second challenge				H5N2 challenge			
**M1**	iButton	40.5 (0.2)	***41.0 (0.3)***	40.9 (0.2)	40.6 (0.1)	40.6 (0.1)	40.6 (0.1)	40.8 (0.2)	40.6 (0.1)	40.6 (0.1)	40.5 (0.2)	40.3 (0.2)	***40.7 (0.1)***
	DSI	40.2 (0.1)	***40.7 (0.2)***	40.7 (0.2)	40.3 (0.1)	40.3 (0.1)	40.3 (0.2)	40.4 (0.1)	40.4 (0.1)	40.3 (0.1)	40.3 (0.3)	40.1 (0.2)	40.4 (0.2)
**M2**	iButton	40.6 (0.2)	***41.1 (0.2)***	40.9 (0.3)	40.7 (0.2)	40.7 (0.2)	40.3 (0.2)	40.6 (0.2)	40.5 (0.2)	40.6 (0.1)	40.4 (0.2)	40.5 (0.2)	40.5 (0.2)
	DSI	40.4 (0.2)	***40.8 (0.2)***	ND	40.6 (0.2)	40.4 (0.2)	ND	ND	ND	40.5 (0.1)	ND	40.5 (0.1)	40.2 (0.1)
**M3**	iButton	40.6 (0.1)	***41.2 (0.3)***	40.9 (0.2)	40.5 (0.2)	40.2 (0.8)	40.2 (0.3)	40.1 (0.4)	40.0 (0.6)	ND	ND	ND	ND
	DSI	40.4 (0.3)	***41.1 (0.3)***	40.8 (0.1)	40.5 (0.2)	40.5 (0.2)	40.5 (0.2)	40.4 (0.3)	40.6 (0.2)	ND	ND	ND	ND
**M4**	iButton	40.5 (0.2)	40.5 (0.1)	***40.9 (0.2)***	41.5 (0.1)	40.8 (0.1)	40.7 (0.1)	40.7 (0.2)	40.7 (0.2)	40.8 (0.1)	40.9 (0.2)	40.6 (0.2)	40.5 (0.3)
	DSI	40.4 (0.2)	40.3 (0.1)	***40.7 (0.2)***	40.9 (0.2)	40.4 (0.1)	40.5 (0.2)	40.6 (0.2)	40.5 (0.3)	40.6 (0.1)	40.7 (0.2)	40.4 (0.3)	40.3 (0.4)
**M5**	iButton	40.7 (0.1)	40.7 (0.2)	40.7 (0.1)	40.8 (0.2)	40.7 (0.1)	40.7 (0.1)	40.7 (0.2)	40.7 (0.2)	40.7 (0.1)	40.7 (0.1)	40.8 (0.1)	40.5 (0.2)
	DSI	40.5 (0.2)	40.4 (0.1)	ND	40.5 (0.2)	40.5 (0.1)	40.6 (0.1)	40.8 (0.2)	40.6 (0.1)	40.6 (0.1)	40.6 (0.1)	40.6 (0.1)	40.3 (0.1)
**M6**	iButton	39.8 (1.4)	40.1 (0.3)	ND	ND	ND	ND	ND	ND	ND	ND	ND	ND
	DSI	40.2 (0.4)	40.2 (0.1)	40.4 (0.1)	40.6 (0.1)	40.5 (0.2)	40.5 (0.2)	40.6 (0.1)	40.6 (0.1)	40.5 (0.1)	40.5 (0.1)	40.5 (0.2)	40.4 (0.1)

Standard deviation is indicated in parentheses. Bold italic values indicate a significant increase (actual day compared with the previous one). ND: no data due to signal out of range (DSI transmitter) or iButton loss.

### Body Mass

**Figure 3:**
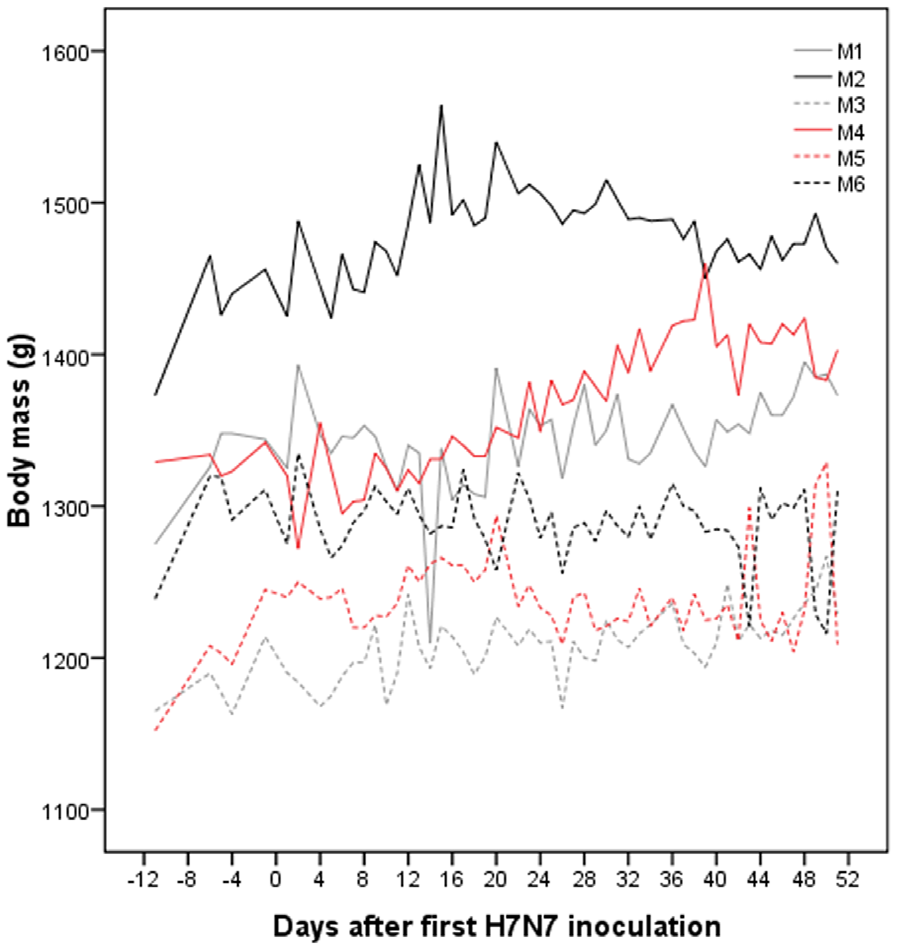
Body mass for each mallard throughout the study.

The six implanted ducks gained weight throughout the study period (paired t-test: t = −12.2, df = 5, p<0.001) with a mean change of 75.2 g (SD = 15.1, Figure 3). Day-to-day comparisons did not reveal an effect of infection on body mass.

### Viral Shedding

Throughout the study period, the total number of samples positive for the matrix gene by real-time reverse transcription polymerase chain reaction (RRT-PCR) was 53 for oral swabs, 65 for water samples, 75 for feces, and 80 for cloacal swabs. The proportion of positive differed among sample types (χ2 = 8.46; df = 3; p = 0.037) and pair-wise comparisons with Bonferroni-corrected p-values showed that oral swabs were less frequently positive than cloacal swabs (χ2 = 7.35; df = 1; p = 0.021).

#### Primary H7N7 inoculation

**Figure 4:**
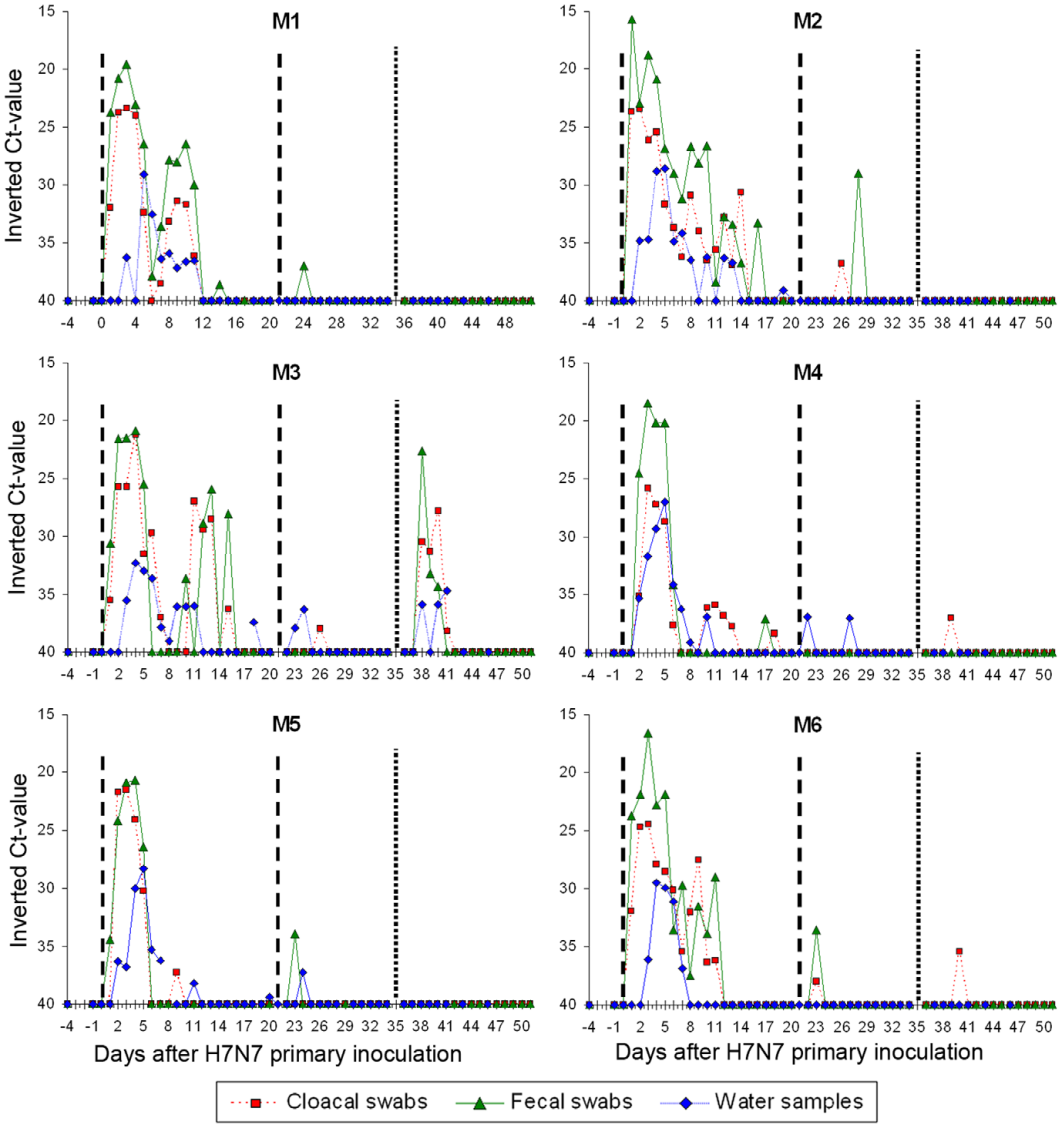
Matrix gene RTT-PCR results for the six implanted mallards. Dash line: H7N7 inoculation, dot line: H5N2 inoculation. Results for oral swabs are presented as supportive material online (Figure S1).

Five of the six ducks excreted viral RNA in their feces on the first day post-inoculation (PI) and all samples (feces, cloacal and oral swabs) from all birds were positive on the second day PI (Figures 4 and S1). Finally, viral RNA was detected in all sample types (fecal, cloacal, oral, and water) three days PI. Considering all sample types, continuous shedding was recorded on average for 12.0 days (SD = 1.1) and intermittent shedding for another 3.7 days (SD = 3.1).

#### Secondary H7N7 inoculation

**Figure 5:**
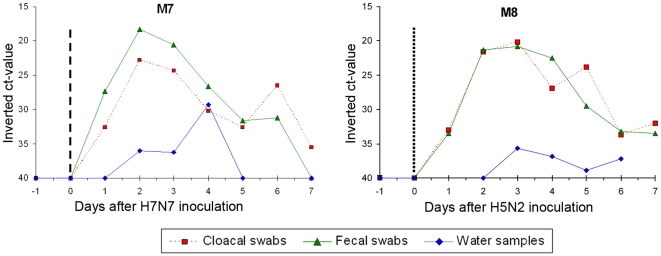
Matrix gene RTT-PCR results for the two control mallards. Dash line: H7N7 infection, dot line: H5N2 infection; euthanasia occurred on day 7 post-inoculation. Results for oral swabs are presented as supportive material online (Figure S2).

Intermittent and moderate (high ct-values) viral RNA shedding was detected for all birds in water, fecal or cloacal samples between day 1 and 7 after H7N7 re-inoculation (Figure 4). Conversely, the control duck (M7) became infected and shed viral RNA with a pattern similar to that observed for the six implanted ducks after H7N7 primary inoculation (Figures 5 and S2).

#### H5N2 inoculation

No viral RNA shedding was recorded for two birds (M1 and M2) whereas low viral RNA excretion (high ct-value) was detected in a single swab for three birds (M4, M6 and M5). These samples were low-positive by the RRT-PCR method targeting the matrix gene but negative by the H5 and H7 RRT-PCR methods (which are less sensitive). It was therefore not possible to determine if the viral RNA excreted by these three ducks was from the H5 or H7 isolate. One duck (M3) excreted H5 viral RNA from day 3 to 6 after H5N2 inoculation (Figures 4 and S1) with a pattern similar to the H5-inoculated control bird (M8) (Figures 5 and S2). All samples from this duck were negative by H7 RRT-PCR.

### Humoral Immune Response

#### Primary H7N7 inoculation

**Figure 6:**
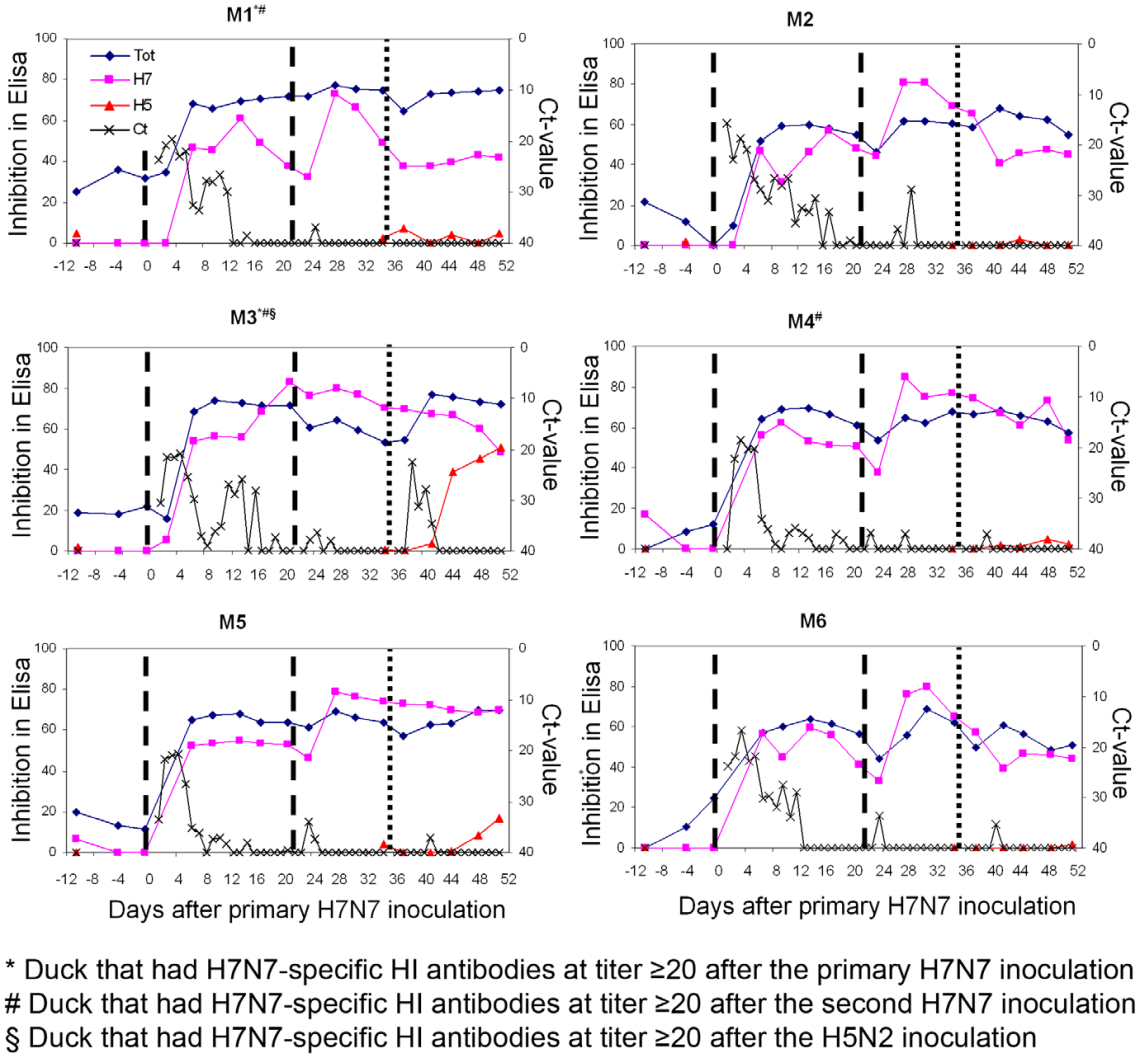
ELISA and matrix gene RTT-PCR results for the six implanted mallards. Primary y-axis: ELISA inhibition values (blue line: NP-ELISA, pink line H7-ELISA and red line: H5-ELISA); secondary y-axis (black line): minimum ct-value considering oral, cloacal, fecal and water samples; the vertical lines illustrate the successive inoculations (dash line: H7N7 inoculation, dot line: H5N2 inoculation).

Antibodies were detected in all birds both by NP- and H7-ELISA one week after the first H7N7 inoculation (Figure 6). H7-specific hemagglutination inhibition (HI) antibodies titers were <20 except for M1 at 13, 16 and 20 days PI (titer 20) and M3 at 20 days PI (titer 40).

#### Secondary H7N7 inoculation

For all birds, H7N7 re-inoculation was followed by an increase in H7- and, to a lesser extent, NP-ELISA inhibition values (Figure 6). H7-specific HI antibodies at titers ≥20 were detected in four birds (M1, M3, M4 and M6).

#### H5N2 inoculation

Antibodies remained detectable by NP- and H7-ELISA in all birds (Figure 6) but only M3 had H7-specific HI antibodies at titers ≥20. H5-specific antibody production was only detected in M3 by H5-ELISA, which was in accordance with the H5N2 viral RNA detection in this bird. H5-specific HI antibodies titers were <20 in all birds.

### Pathology

The six implanted ducks (M1-M6) were euthanized at the end of the study (51 days PI) whereas the two control ducks (M7 and M8) were euthanized 7 days post-challenge. No lesion potentially associated with LPAIV infection was detected grossly or histologically in any of the ducks and no IAV antigen was detected by immunohistochemistry in any of the tested organs. Two birds had a mild proctitis and colitis with infiltration of mononuclear cells (primarily lymphocytes) in the lamina propria, likely a result of the presence of spirochetes. Varying amounts of spirochetes were seen in the caecum of all mallards, which confirmed the positive screening previously observed on feces. A subcutaneous granulomatous inflammation was observed around the transponder in four birds.

## Discussion

### Impact of LPAI Infection

The overall response to infection was minimal. All six implanted mallards remained alert with neither clinical nor pathological signs of disease both after the LPAIV H7N7 primary inoculation and after the successive re-inoculations. Their activity (i.e. movements per minutes) monitored by DSI transponders was not modified after inoculation and heart rate values remained in agreement with mean values reported for mallards [20]. Only a brief (∼2 days) and small (∼0.5°C) increase in body temperature was recorded in four of the implanted birds on the day they started shedding viral RNA in their feces. Because fever is known to be monophasic in the closely related Pekin duck [21], it is possible that the four individuals which showed an increased body temperature developed a slight short-term fever during the H7N7 primary infection.

The minimal response detected in this study does not allow concluding whether infection with LPAIVs may have a significant impact on mallard populations. Ecologic studies in a natural environment are needed to assess if the increase in body temperature observed during early infection is associated with ecological costs. Developing fever and mounting an immunologic response are physiologically costly and, in situations in which animals are resource limited and exposed to predators, these costs may have to be balanced against other expenses such as reproduction, growth, molt, or migration [22], [23].

Previous studies reported that female mallards experimentally infected with a LPAIV isolate significantly decreased egg production during the following week [24] and field investigations showed that LPAIV infected mallards have a lower body mass than negative mallards [19]. A correlation between IAV infection and migration success has also been suspected in Bewick swans [25] but field investigations on mallards captured in southeast Sweden failed to show a correlation between infection and migration speed or distance [19].

### Homosubtypic and Heterosubtypic Immunity

During autumn migration, juvenile mallards get in contact with congeners coming from other areas and may successively be exposed to various IAV subtypes [7], [12]. This study had a design to reflect these relatively short-term (i.e. within season) re-exposures. Because the samples were only tested by RRT-PCR, it is not known for how long infective viral particles were excreted but we may speculate from previous studies that viral isolation would likely have been successful for samples with low ct-values (i.e. ct-values <30) [26], [27]. Positive oral swabs and water samples were likely the result of an environmental contamination by viral particles shed in feces.

Homologous re-inoculation of the H7N7 isolate 21 days after primo-inoculation induced a weak secondary antibody response and the shedding pattern was very different from that observed after primo-inoculation, with only intermittent detection of viral RNA in feces, pool water or cloacal swabs. As previously reported in experimental infections of mallards with LPAIV strains, HI antibody titers were low, confirming that ELISA sensitivity exceeds that of HI test and that protection against homologous re-infection exists despite the absence of significant HI titers.

Heterologous challenge with an H5N2 isolate from a wild mallard was performed 14 days after the homologous H7N7 re-inoculation. Active H5 infection was confirmed only in one duck, by expression of H5-specific antibodies and detection of viral RNA in the various sample types (feces, water, oral and cloacal swabs) with a pattern similar to the H5-inoculated control bird. In all other ducks, H5-specific antibodies were not detected and no or only intermittent viral RNA shedding was observed after the heterologous challenge. In these ducks, prior infection with H7N7 LPAIV seems to have provided protection against infection by H5N2 LPAIV. This result supports speculations from Sharp et al. [28] who suggested that, if infections with two well-adapted heterosubtypic viruses occur at different times, the first infectant may prevent the replication of a later infectant.

Another study on mallards recently reported a protective effect of infection by an H4N6 LPAIV strain prior to exposure to an HP H5N1 [18]. Further investigations with other viral strains and different challenge timings are needed to better assess the importance of heterologous immunity in ducks. In chickens, gut immune responses induced by exposure to LPAIVs are suspected to play an important role in protection from the lethal effects of HPAI strains [29]. Likewise, local immunity may influence the outcome of duck infections and provide protection against further infections. In mallards, HI antibodies were detected in the bile and peaked at about 12 days PI, i.e. approximately the moment when cloacal shedding ceased [30]. Additionally, cellular immune response was shown to play a role in the protection of chickens [31] and turkeys [32] against heterologous infections and may be important in the protection of ducks [29], [33]. Inter-individual variations in immunity also likely exist, as revealed in our study by replication of the heterologous re-infectant virus in one duck.

Because the heterologous challenges were separated by a short interval (2 weeks), RNA excretion may have been influenced both by the host protective immune responses and by the impact of co-infection on virus replication. Further investigations about viral interactions, cellular and local immunity are needed to understand when heterologous re-exposure leads to co-infection compared to when elimination of the re-infectant occurs. Because concomitant infections may lead to genetic reassortment and emergence of new viral strains, these studies are essential in understanding the overall ecology of AIVs.

### Conclusions

This study showed that in mallards (1) a mild transient increase in body temperature may occur during LPAIV infection, (2) infection by a LPAIV is limited by prior infection with a homologous strain and (3) may be prevented by prior infection with a heterologous strain. The study also showed that (4) individual heterogeneity exists, both in the susceptibility to infection and the ability to develop heterosubtypic immunity. Finally, the study showed that (5) viral RNA intermittent shedding occurs and that (6) the daily shedding pattern differs among sample types (water, feces, cloacal and oral swabs).

Further investigations are needed to better assess the ecological costs of IAVs on wild waterfowl populations because such costs may deeply influence the transmission dynamics of the viruses [10]. Field studies based on frequent sampling of the same individuals are encouraged to gain a better knowledge on how frequently re-infections occur in nature and how they influence viral shedding patterns. Experimental infections with longer time intervals between challenges (e.g. several months) would also be useful to investigate long-term immunity. Such data will be essential to optimize virus transmission models and better understand IAV ecology in their natural hosts.

## Materials and Methods

### Ethics Statement

The animal experiment procedures were approved by the Committee for Laboratory Animal Science of the Swedish Board of Agriculture.

### Animals

Eight 3-month-old male wild-type mallards (*Anas platyrhynchos*) of approximately the same body mass and size (measurement of the left wing, the right tarsus length and the distance from bill tip to back of the skull) were selected from a Swedish duck farm known from previous successive sampling to be free from IAV infection. The eight mallards were isolated from the other ducks for ten days before they were transferred to the animal house.

Absence of active shedding of influenza viruses was checked three times, at one-week intervals, by testing oral and cloacal swabs using RRT-PCR (see below for methods). Absence of previous exposure to influenza viruses was also checked by NP-ELISA on sera sampled three successive times at one-week-intervals. The eight mallards were also checked for the presence of intestinal pathogens known to occur in mallards, i.e. intestinal parasites [34], coronaviruses [35], *Chlamydophila* sp. [36], and *Brachyspira* sp. spirochetes [37]. They were free from infection, except for *Brachyspira* sp., which were found in all ducks. Culture of fecal material showed that the bacterial flora were normal [34].

The ducks were individually identified by unique color combinations of plastic rings placed around their right tarsus and randomly separated in two groups (Figure 1). The six mallards assigned to the first group (referred to as M1, M2, etc… M6) were surgically implanted with data loggers (see below) and successively infected with two strains of IAVs. The other two ducks (M7 and M8) were used as controls for virus infectivity during a 7-day-period.

### Housing

The study took place within the animal facility of the Swedish Institute for Infectious Disease Control (SMI) in Stockholm, Sweden. The mallards were kept in individual cages with access to an individual pool and a shelter and were fed an equal mixture of chicken food and crushed wheat and oat *ad libitum*. The cages were cleaned and the water from each pool changed every morning.

All ducks were caged in the same room, except the controls for virus infectivity which were kept separately until the day they were challenged: M7 was introduced in the room on day 21 (and euthanized on day 28) whereas M8 was introduced in the room on day 35 (and euthanized on day 42).

### Radiotelemetry

A DSI transmitter (Data Sciences International, model TA11-CTA-F40), consisting of a plastic body and two electrodes, was surgically implanted under the skin of six ducks (M1 to M6). Twice every minute during the whole study period, the DSI transmitter stored data on body temperature (°C), heart rate (beats per minute) and activity (movements per minute of the implanted device relative to the receiver) in a computer using Dataquest A.R.T. Data Acquisition Software, Gold Package. Each transmitter was in contact with a receiver (model RPC-1) placed under the cages, and all six receivers were connected through two data exchange matrixes connected to the computer. Details about DSI data acquisition system can be found in Savory and Kostal [38]. A preliminary study using lipopolysaccharide (as in Maloney and Gray [21]) revealed that the system allowed detection of increased body temperature and heart rate (Jourdain et al., unpublished data).

As previously reported, handling of the ducks resulted in increased body temperature [21] and heart rate [20], and body temperature was lower when the light was off [39]; therefore, only data recorded during this time (7.00 pm to 6.00 am) were used in the analyses. In total, 76,061 data points were analyzed for each of the three DSI parameters (N_M1_ = 14099; N_M2_ = 7224; N_M3_ = 13837; N_M4_ = 27557; N_M5_ = 4668; N_M6_ = 8676;), corresponding to only 34% of the recording potential of the system. The remaining 66% of data were not recorded due to signal loss (i.e., cage area was larger than receiver range, which was ∼40 cm).

### Thermochron iButtons

Because DSI transmitters have not been used on mallards before, and to cover possible technical failures, a Thermochron iButton (Maxim Integrated Products; model DS1922L), which records temperature into an internal memory [40], was also surgically implanted under the skin of the six transmitter-implanted birds (M1 to M6). The iButtons were programmed (using 1-wire Drivers software, version 4.00) to measure body temperature (°C) every half hour throughout the study period. The iButtons were recovered from the ducks after euthanasia and data were downloaded using 1-wire Drivers software. An iButton placed in the experiment room monitored the room temperature at the same frequency as for the duck body temperature and showed that room temperature was stable throughout the experiment (21.5°C, SD = 0.1).

In total, the iButtons recorded 6,216 body temperature data points, i.e. about 1,300 data points for each duck except M3 (N = 794) and M6 (N = 222) which lost their respective iButtons before the end of the experiment (days 28 and 2 PI, respectively).

### Anesthesia and postoperative care

Surgery under general anesthesia was performed on six ducks (M1 to M6) to equip them with a DSI transmitter and an iButton. Food was withdrawn the night before surgery to avoid false deglutition during anesthesia. Before cutting the feathers from the incision areas, each duck was sedated by intramuscular administration of meloxicam (Metacam®, 0.5 mg/kg), butorphanol (Turbogesic®, 1 mg/kg) and xylazine (Paxman®, 1 mg/kg). Anesthesia was induced using 3–4% isoflurane (Forene®) with an oxygen flow of 2–3 L/min administered via a small-dog narcomask. A local anesthetic cream (EMLA®) was applied on the different incision sites and an eye gel (Lubrithal®) was applied in both eyes. During surgery, 2–2.5% isoflurane was administered with an oxygen flow of 1–2 L/min. After surgery, an oxygen flow of 4 L/min was used. Once awake, the duck was placed back into its cage under an infrared light. To prevent infection of the wounds, a long-lasting antibiotic (amoxicillin LA, Vetrimoxin®, 15 mg/kg) was injected in the pectoral muscle both after surgery and two days later. Meloxicam (0.5 mg/kg) was re-administrated one day after surgery to minimize inflammation. Access to the pool was prevented until 2–3 days after surgery.

### Surgery

The body of the transponder and the electrocardiogram leads were positioned in a base-apex configuration as described by Harm and colleagues [41]. Body feathers above and around the incision areas were cut with bended scissors. Incision areas were then cleaned and disinfected with povidone iodine, and an anesthetic cream was applied. A dorsal skin incision of approximately 3 cm was made at the base of the neck, between the shoulders. The iButton was inserted through this incision and, after blunt dissection, was placed subcutaneously on the left part of the back. The body of the DSI transmitter was inserted through the same incision and attached to the skin by two stitches using non-absorbable surgical suture (Supramid 3/0, Braun). A second incision was made at the base of the right wing. The negative electrode was then tunneled with a trocar until it reached the wing incision. It was fixed to the muscular fibers by 3–4 stitches of absorbable surgical suture (Vicryl 3/0, Ethicon). The positive electrode was similarly fixed to the left *Pectoralis major* muscle after being tunneled to the abdomen. All skin incisions were sewn up with an absorbable surgical suture (Vicryl rapid 3/0, Ethicon). The ducks were allowed to recover from surgery for at least 10 days prior to starting the monitoring of individual data (Figure 1).

### Experimental Design

The experiment was divided into four successive periods, during which the six implanted mallards were monitored continuously (body temperature, heart rate, activity) and weighed and sampled daily (Figure 1). The first period (1 week) allowed monitoring baseline body temperature, heart rate and activity levels for each mallard. The second period (3 weeks) aimed at studying the effects of primo-infection with an H7N7 LPAIV strain inoculated in the esophagus (10^8.7^ EID_50_ in a 1 mL inoculum). This three-week-period corresponds to the maximum time during which IAVs are usually excreted by infected ducks [3], [17], [30], [42]. The third period (2 weeks) investigated the impact of re-inoculation with the same H7N7 LPAIV strain administered through the same route and at the same dose. A naïve mallard (M7) was simultaneously inoculated (through the same route and at the same dose to serve as a positive control) and necropsied 7 days later to search for lesions associated with infection by the H7N7 isolate. The fourth period (2.5 weeks) allowed studying the effects of heterologous inoculation in the esophagus with an LPAIV H5N2 strain (10^8.7^ EID_50_ in a 1 mL inoculum). As previously, a naïve mallard (M8) was used as a positive control. It was inoculated along with the other ducks and necropsied 7 days later to search for lesions associated with infection by the H5N2 isolate. The six implanted ducks were euthanized 51 days after the first inoculation and necropsied.

### Virus Preparation

Two LPAIV strains isolated in 2004 from wild mallards at Ottenby, Southern Sweden, were used: A/mallard/Sweden/7206/2004 (H7N7) and A/mallard/Sweden/6566/2004 (H5N2). New viral stocks were grown by inoculating 200 µL of the selected isolates (dilution 1:50 in PBS) in the allantoic cavity of 10-day-old embryonated chicken eggs. The corresponding allantoic fluid was harvested three days later, centrifuged and pooled. Viral titers were determined by 50% Embryo Infectious Dose (EID_50_) using the method of Reed and Muench [43].

### Sampling

Water samples, feces, oral and cloacal swabs were collected daily and blood samples bi-weekly throughout the study, from day −7 to 51 (Figure 1). Every morning, before the cages were cleaned, 40 mL of water was sampled from each pool and stored directly at −80°C. The mallards were placed in individual single-use paper boxes for a few minutes before being sampled. They were swabbed from the cloaca and oral cavity and fecal samples were collected by rolling a sterile cotton swab in the fresh droppings left in the paper box. The swabs were placed in 1 mL of virus transportation medium (Hanks balanced salt solution) as described in Wallensten et al. [7] and kept on ice until they were stored at −80°C. The ducks were bled biweekly, alternating between the right and left brachial veins, for serological analyses. After centrifugation, sera were stored at −20°C.

Biosafety precautions were used between handling the ducks by spraying the gloves, table and lab coats with an alcoholic solution. Before their inclusion in the study (on day 21 and 35, respectively), the control ducks M7 and M8 were handled before the other ducks and in a separate room.

### Real-Time Reverse Transcription Polymerase Chain Reaction (RRT-PCR)

#### Matrix gene RRT-PCR for fecal samples, cloacal and oral swabs

After thawing, the tubes were thoroughly vortexed and 150 µl were removed and mixed with 450 µl Trizol reagent (Invitrogen, Paisley, UK) for virus inactivation. Cold chloroform (160 µl) was added to yield an excess of 300 µl needed for RNA extraction. After vortexing, the water and organic phases were allowed to separate for 1–2 minutes after which the tubes were centrifuged at 14000 g for 15 minutes. The water phase (300 µl) was then removed and RNA extracted using the M48 Biorobot (Qiagen, Hilden, Germany) with the MagAttract Viral RNA M48 extraction kit (Qiagen) according to the manufacturer's specifications and eluted in 65 µl. A RRT-PCR system targeting the matrix gene was used for screening and quantification [44]. The PCR was run in a one-step fashion using QuantiTect Probe RT-PCR kit (Qiagen) according to the manufacturer's specifications for ABI 7900HT PCR machine (Applied Biosystems, Carlsbad, USA) and 5 µl RNA was used as template. Cycle threshold (ct-) values obtained were normalized by setting threshold value to 0.1 for all runs. A ct-value of <40 was considered positive for LPAIV antigen.

#### Matrix gene RRT-PCR for water samples

A slightly different protocol was used for water samples. After thawing, the tubes were thoroughly vortexed and 400 µl were removed for direct RNA extraction using the MagAttract Viral RNA M48 extraction kit with the BioRobot M48 set to obtain 75 µL of elution volume. A LightCycler 1.5 (Roche Diagnostics GmbH, Germany) was used to perform the thermo-cycling. A ct-value of <40 was considered positive for LPAIV antigen.

#### H5 and H7 RRT-PCR

Only water and swab samples collected from the implanted ducks after day 35 (H5N2 inoculation) and positive for the matrix gene (n = 13) were further tested for the presence of H5 or H7-specific viral RNA. RNA was extracted as before and RRT-PCR performed using a Taqman probes system as described by EU Community Reference Laboratory protocols [45].

### Serology

Serum samples were tested for antibodies targeting IAV nucleoprotein (NP) with a commercial ELISA kit (NP-ELISA, Pourquier Avian Influenza A Blocking ELISA, Montpellier, France). The presence of H7- and H5-specific antibodies was studied by using the ID Screen Influenza H7 Antibody Competition and ID Screen Influenza H5 Antibody Competition (ID VET Innovative Diagnostics, Montpellier, France). According to the manufacturers' instructions, sera were considered positive if the calculated value was ≥65% (NP-ELISA) or ≥50% (H7- and H5-ELISA).

In order to detect HI antibodies specific for H5 and H7 respectively, HI test was performed following standard procedures [11], using red blood cells from specific pathogen-free chickens and four HA units of the viral strains used for the experimental challenge. Only samples with a titer ≥20 were considered.

### Pathology and Immunohistochemical Testing

The two mallards that served as positive controls (M7 for H7N7 infection and M8 for H5N2 infection) were euthanized 7 days PI with H7N7 or H5N2, respectively, whereas the six implanted mallards were euthanized at the end of the study (51 days PI). The birds were euthanized with 100 mg/kg sodium pentobarbital (Pentobarbital vet®, 100 mg/ml i.v.). Routine necropsies were carried out directly after euthanasia. Tissue samples (brain, lungs, trachea, air sacs, heart, liver, spleen, kidneys, pancreas, adrenal glands, duodenum, jejunum, colon, caecum, ventriculus, proventriculus, and testicles) were fixed in 10% neutral buffered formalin for histopathology and immunohistochemistry (IHC).

After formalin fixation, the tissue samples were processed routinely, sectioned at 4–5 µm, stained with hematoxylin and eosin, and examined for pathologic changes. In order to investigate the presence of viral antigen, duplicate sections of liver, lung, trachea, air sacs, pancreas, duodenum, jejunum, colon, and caecum from all ducks, as well as all other collected tissues from the two control ducks, were processed for IHC using a commercial anti-influenza A nucleoprotein primary monoclonal antibody [46]. Each immunostain included a positive reference control and a negative control. Each section was also accompanied by a primary antibody–omitted control. Sections from all levels of intestine were further stained with Warthin-Starry silver stain in order to investigate the presence of spirochetes.

### Statistical Analyses

Statistical analyses of the physiological parameters were done individually for each implanted mallard. Multivariate General Linear Models (GLMs) were used with mean values for each hour and day of DSI body temperature, activity, and heart rate as dependent variables, and day and hour as independent variables. The latter variable was included to control for the fact that the physiological variables varied over time. Additional univariate GLMs were run with iButton body temperature as the dependent variable and the same predictors as above. For three ducks (M1, M3 and M4), data were transformed to obtain normally distributed residuals. Day was a significant variable (p<0.001) for all ducks and dependent variables (except for the heart rate of M2). Because it was biologically relevant to compare values obtained on successive days, we used Tukey's post hoc test to compare the means from the control period with the means from day-one-PI, day-one-PI with day-two-PI, etc… until the end of the experiment.

Body mass changes were analyzed on a daily basis by comparing each day with the previous one as described above. Because body mass is naturally dependent on preceding values, paired t-tests with Bonferroni-corrected p-values were used. In addition, paired t-tests were used to get an overview of how the body mass of each duck changed over the study period.

In order to show whether there was any difference in virus detection between the four sample types (oral and cloacal swabs, feces and pool water), the proportion of positive samples for the different techniques was analyzed with χ2-statistics. Only days for which all sample types from a given duck had been tested were included in this analysis.

## Supporting Information

Figure S1 Matrix gene RTT-PCR results for oral swabs from the six implanted mallards. Dash line: H7N7 inoculation, dot line: H5N2 inoculation
(7.76 MB TIF)

Figure S2 Matrix gene RTT-PCR results for oral swabs from the two control mallards. Dash line: H7N7 infection, dot line: H5N2 infection; euthanasia occurred on day 7 post-inoculation
(3.00 MB TIF)
