# Supplementary figures and images for: Influenza Virus in a Natural Host, the Mallard: Experimental Infection Data
Source: PLoS One. 2010 Jan 28;5(1):e8935. doi: 10.1371/journal.pone.0008935 (PMC2812492; doi:10.1371/journal.pone.0008935)

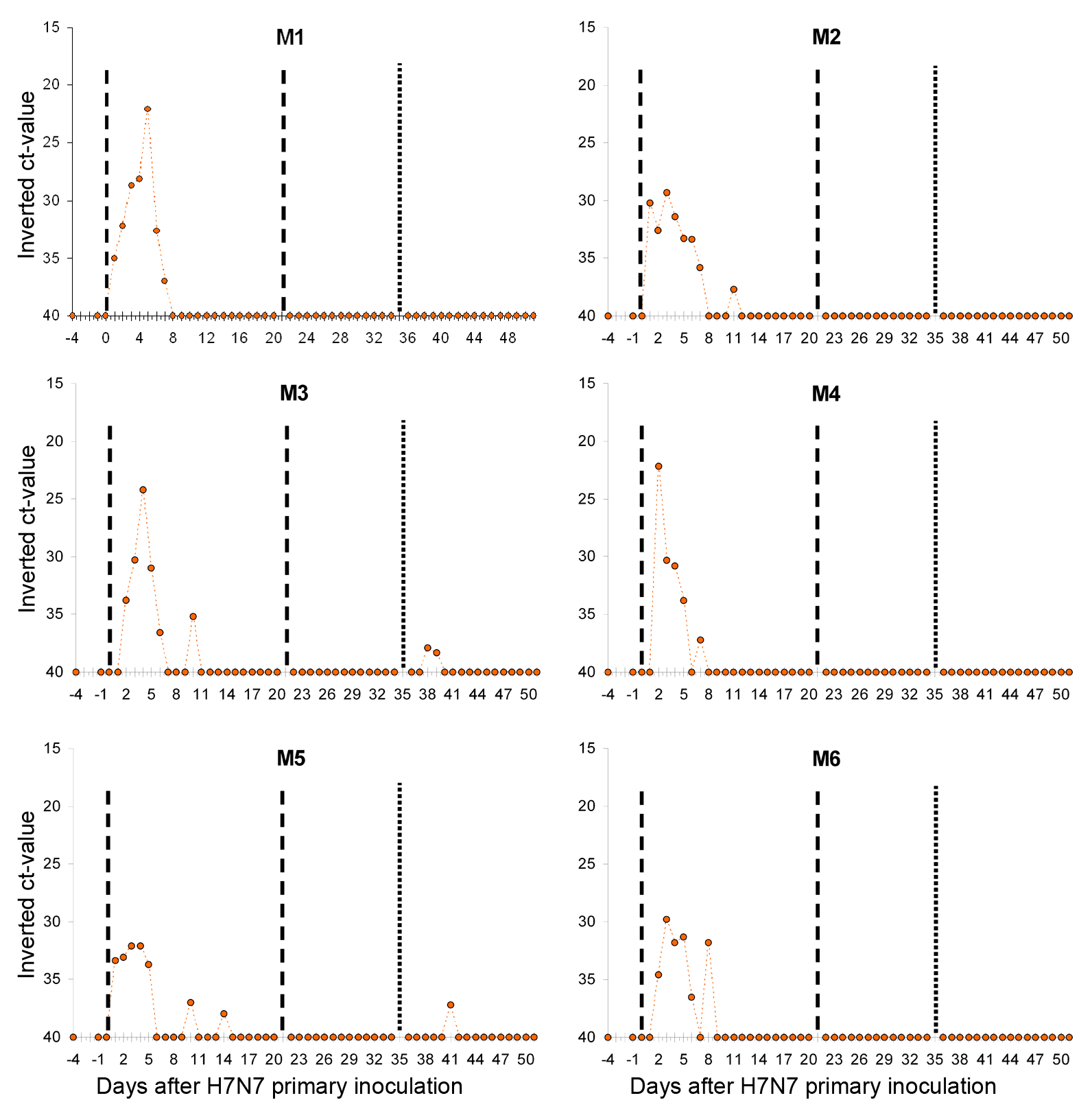

Supplement: Figure S1 — Matrix gene RTT-PCR results for oral swabs from the six implanted mallards. Dash line: H7N7 inoculation, dot line: H5N2 inoculation (7.76 MB TIF) [file pone.0008935.s001.tif]

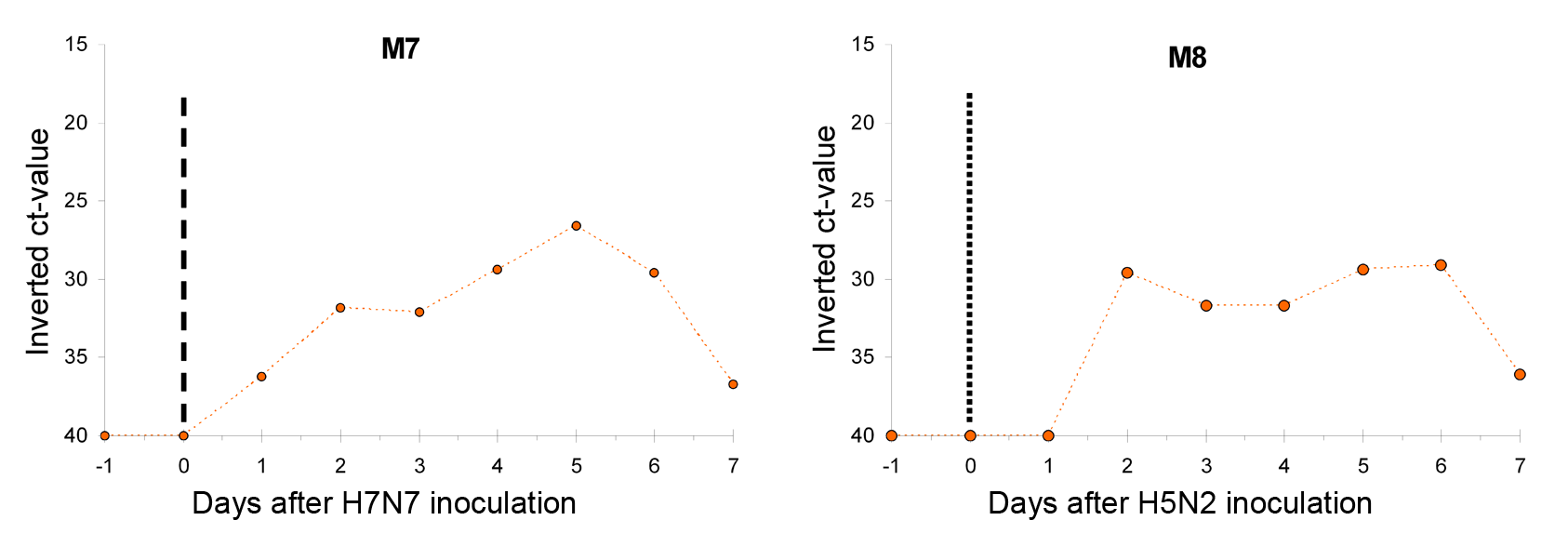

Supplement: Figure S2 — Matrix gene RTT-PCR results for oral swabs from the two control mallards. Dash line: H7N7 infection, dot line: H5N2 infection; euthanasia occurred on day 7 post-inoculation (3.00 MB TIF) [file pone.0008935.s002.tif]
